# Separation of ^44^Sc from ^44^Ti in the Context of A Generator System for Radiopharmaceutical Purposes with the Example of [^44^Sc]Sc-PSMA-617 and [^44^Sc]Sc-PSMA-I&T Synthesis

**DOI:** 10.3390/molecules26216371

**Published:** 2021-10-21

**Authors:** Anton A. Larenkov, Artur G. Makichyan, Vladimir N. Iatsenko

**Affiliations:** State Research Center–Burnasyan Federal Medical Biophysical Center of Federal Medical Biological Agency, Zhivopisnaya Str., bld. 46, 123098 Moscow, Russia; makbezh@gmail.com (A.G.M.); vlad-yatsenko@mail.ru (V.N.I.)

**Keywords:** scandium-44, ^44^Ti/^44^Sc radionuclide generator, TEVA resin, Presep PolyChelate resin, radiopharmaceuticals, [^44^Sc]Sc-PSMA-617, [^44^Sc]Sc-PSMA-I&T

## Abstract

Today, ^44^Sc is an attractive radionuclide for molecular imaging with PET. In this work, we evaluated a ^44^Ti/^44^Sc radionuclide generator based on TEVA resin as a source of ^44^Sc. The generator prototype (5 MBq) exhibits high ^44^Ti retention and stable yield of ^44^Sc (91 ± 6 %) in 1 mL of eluate (20 bed volumes, eluent—0.1 M oxalic acid/0.2 M HCl) during one year of monitoring (more than 120 elutions). The breakthrough of ^44^Ti did not exceed 1.5 × 10^−5^% (average value was 6.5 × 10^−6^%). Post-processing of the eluate for further use in radiopharmaceutical synthesis was proposed. The post-processing procedure using a combination of Presep^®^ PolyChelate and TK221 resins made it possible to obtain ^44^Sc-radioconjugates with high labeling yield (≥95%) while using small precursor amounts (5 nmol). The proposed method takes no more than 15 min and provides ≥90% yield relative to the ^44^Sc activity eluted from the generator. The labeling efficiency was demonstrated on the example of [^44^Sc]Sc-PSMA-617 and [^44^Sc]Sc-PSMA-I&T synthesis. Some superiority of PSMA-I&T over PSMA-617 in terms of ^44^Sc labeling efficiency was demonstrated (likely due to presence of DOTAGA chelator in the precursor structure). It was also shown that microwave heating of the reaction mixture considerably shortened the reaction time and improved radiolabeling yield and reproducibility of [^44^Sc]Sc-PSMA-617 and [^44^Sc]Sc-PSMA-I&T synthesis.

## 1. Introduction

The advantages of nuclear medicine methods have led to its sustainable development over the past few decades and its transformation into an integral part of clinical practice in developed countries. The active introduction of positron emission tomography (PET) and radionuclide therapy (RNT) in medical institutions, together with constantly developing new radiopharmaceuticals (RP), has made it possible to raise the possibilities of diagnostics and therapy in a variety of socially significant diseases to new levels.

^68^Ga (T_½_ = 67.83 min., β^+^—88.9%, E_β_^max^ = 1899.1 keV [1]), obtained from the ^68^Gе/^68^Ga radionuclide generator, is the most actively used radiometal for synthesis of PET radiopharmaceuticals in the world today. For all its merits, its short physical half-life limits the available imaging period to just a few hours. PET scans at later times after injection (when the contrast of images increases due to biodistribution), as well as reliable dosimetry calculations and therapy planning (for long-lived therapeutic radionuclides, such as ^177^Lu) cannot be performed with gallium-68. In the light of these considerations, the interest of researchers in scandium-44 has increased [2].

^44^Sc (T_½_ = 3.97 h, β^+^—94.3%, E_β_^max^ = 1474.3 keV [1]) has a physical half-life almost four times longer than ^68^Ga. This fact allows for imaging in a longer time interval (up to 24 h after administration), which increases the efficiency of lesion imaging by increasing the target to nontarget ratio. Clinical studies have demonstrated that PET/CT with [^44^Sc]Sc-DOTATOC 4 h after injection allows the detection of tumor foci that were not visualized with [^68^Ga]Ga-DOTATOC [3]. The mean positron energy of ^44^Sc (Ē_β_—0.63 MeV) is lower than that of ^68^Ga (Ē_β_—0.83 MeV), which leads to better spatial image resolutions of ^44^Sc-PET [4,5,6]. Additionally, the coordination chemistry of ^44^Sc is similar to that of ^90^Y and lanthanides, so it forms stable radioconjugates with a similar structures and, apparently, similar receptor affinities [7]. Thus, ^44^Sc is a very promising radiometal with significant practical potential that may be used in preclinical and clinical PET studies, and most importantly, as a diagnostic match to therapeutical radionuclides of rare-earth elements (e.g., ^47^Sc, ^90^Y, ^153^Sm, ^161^Tb, ^177^Lu) in a theragnostic approach [8]. The results of clinical applications of [^44^Sc]Sc-DOTATOC [3] for the diagnosis of neuroendocrine tumors, as well as [^44^Sc]Sc-PSMA-617 [9,10] for the diagnosis of prostate cancer have been published. It is hypothesized, however, that the clinical application of ^44^Sc may be compromised by the high dose exposure caused by the co-emission of 1157 keV γ-rays (99.9%) [11]. These concerns of radiation safety may be addressed by using tungsten-based containers that are employed for other commercial PET nuclides with high energy γ-emission (such as ^89^Zr). On the other hand, this γ-co-emission of ^44^Sc could be relevant to a new imaging approach based on the three-dimensional measurement of the emitter location using β^+^/γ emitters [8,12].

^44^Sc can be obtained by both using a cyclotron from a calcium target [13,14,15] and using a radionuclide generator from the parent radionuclide ^44^Ti (T_½_ = 60.0 a [1]). Regarding the potentially wide clinical application of ^44^Sc (up to the current level of ^68^Ga use), the cyclotron method of production is preferable, considering the cross sections and yields of ^44^Ca(p,n)^44^Sc and ^45^Sc(p,2n)^44^Ti→^44^Sc reactions [16]. At the same time, a ^44^Ti/^44^Sc generator could be a useful tool for universities and research centers wishing to start working with ^44^Sc but without a cyclotron of their own, and/or those who are far enough from suitable accelerators. Based on the ratio of the decay constants of ^44^Ti (λ_1_ = 3.66 × 10^−10^·s^−1^) and ^44^Sc (λ_2_ = 4.85 × 10^−5^·s^−1^), the maximum time required to reach the secular equilibrium in this pair is ∼67.5 h. At the same time, after 4 h, the level of accumulation of the daughter radionuclide is already 50%, after 12 h—87.7%, after 24 h—98.5%, and after 40 h—99.9 %. Thus, in the presence of a sufficiently effective separation method for ^44^Ti and ^44^Sc, characterized by low ^44^Ti breakthrough and a high ^44^Sc yield, the separation of the daughter radionuclide for research purposes can be carried out daily. The half-life of ^44^Ti, in turn, provides, in theory, an extremely long possible generator operation period.

The earliest published (and probably the first) work about ^44^Ti/^44^Sc generators belongs to Margaret Greene [17], who, based on data on the anion-exchange separation of scandium, vanadium, and titanium [18], suggested using Dowex 1×8 resin as a sorbent for ^44^Ti and a mixture of 0.1 M oxalic acid in 0.2 M HCl for ^44^Sc elution. The yield of ^44^Sc was 60–70%, while the breakthrough of titanium-44 was at the level of 0.02% and increased to 0.1% after 40 elutions. Later, different prototypes of ^44^Ti/^44^Sc generators were described using hydrous zirconium oxide [19], Bio-Rad AG 1×8 anion-exchange resin [20], and ZR hydroxamate resin [21] as a sorbent (see Discussion). The generator presented by Filosofov et al. [20] has the highest load of titanium-44 to date (185 MBq). The yield of ^44^Sc is 97% in 20 mL of eluate, which is a mixture of 0.005 M oxalic acid/0.07 M HCl (preconcentration of activity for further radiolabeling is required), and for the stability of the sorption front of titanium-44 it is necessary to use reverse elution. This generator was used for the first in-human study of [^44^Sc]Sc-PSMA-617 for PET imaging of metastasized castrate-resistant prostate cancer [10].

This article is devoted to the evaluation of a ^44^Ti/^44^Sc generator prototype based on Greene’s concept [17], but using TEVA resin (**TE**tra**V**alent **A**ctinides; quaternary ammonium salt, also called Aliquat^®^ 336 as extractant) [22,23]. Evaluation of suitability of the obtained scandium-44 solutions for radiolabeling was carried out using PSMA-617 and PSMA-I&T precursors (Figure S1).

## 2. Results

Two generator columns (PEEK 100 mm × Ø2.1 mm, 150 mg of TEVA resin) with an initial load of 0.5 and 5.0 MBq ^44^Ti were prepared for this study and used throughout the work. During the study, several additional generator columns with ∼1.5 MBq ^44^Ti were prepared. They were used to evaluate and optimize the separation parameters of ^44^Ti and ^44^Sc in dynamic conditions, as well as to collect data for statistics, but they have not been operated for a long time. This work was started with one tentative experiment as proof-of-concept for Greene’s method [17] with 0.1 M H_2_C_2_O_4_/0.2 M HCl as eluent but with TEVA resin instead of Dowex 1 × 8 as sorbent. The very first generator prototype with an initial ^44^Ti load of 0.5 MBq demonstrated 90% ^44^Sc yield and ≤5.5 × 10^−5^% of ^44^Ti breakthrough. The results obtained made it possible to continue further work, starting with the determination of the distribution coefficients of ^44^Ti and ^44^Sc on TEVA resin.

### 2.1. Distribution Coefficients of ^44^Ti and ^44^Sc on TEVA Resin in HCl-Oxalic Acid Media

Results of the Dg values obtained for ^44^Ti and ^44^Sc on TEVA resin in HCl-oxalic acid media are shown in Table 1. Since we took the Greene’s concept as a basis, attention was mainly paid to the oxalic acid concentration of 0.1 M and various concentrations of hydrochloric acid. The determination of the distribution coefficients of ^44^Ti and ^44^Sc in pure 4 M HCl was carried out to assess the possibility of ^44^Ti recovery from the resin. Blends of 0.005 M H_2_C_2_O_4_/0.007 M HCl and 0.025 M H_2_C_2_O_4_/0.125 M HCl presented by Filosofov et al. [20] have been added for comparison.

**Figure 1:**
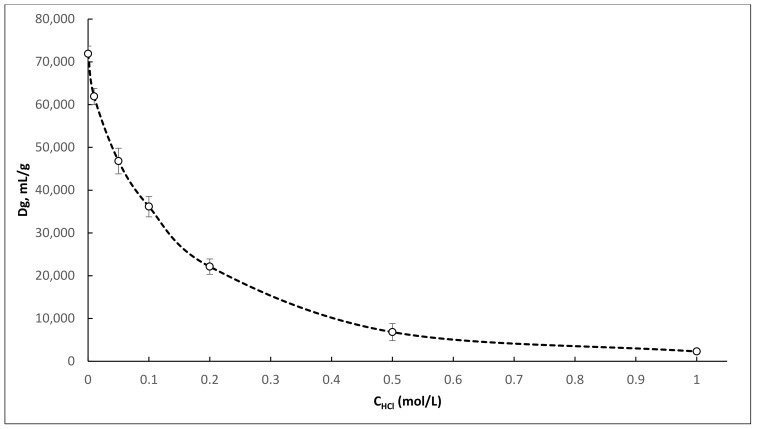
^44^Ti distribution coefficients dependencies on HCl concentration in a mixture of 0.1 M H_2_C_2_O_4_ for TEVA resin.

Obtained distribution coefficients for ^44^Ti on TEVA resin in a mixture of 0.1 M oxalic acid with 0.01–1.0 M HCl (Figure 1) are significantly higher than those presented by McAlister [24] for Ti on TEVA resin in a mixture of 0.05 M H_2_C_2_O_4_/0.01–1.0 M HCl. However, the obtained dependences have a similar form and correlate well with each other. Based on the obtained Dg values, the mixtures of 0.1 M H_2_C_2_O_4_/0.05–0.2 M HCl, and 0.025 M H_2_C_2_O_4_/0.125 M HCl are the most suitable for the separation of ^44^Ti and ^44^Sc. These mixtures were selected for further study under dynamic conditions.

### 2.2. Dynamic Column Separation Experiments

The results of ^44^Ti and ^44^Sc separation under dynamic conditions for the selected eluents are presented in Table 2 (a mixture of 0.005 M H_2_C_2_O_4_/0.007 M HCl was also added for comparison). For each selected composition, a separate column with a load of ∼1.5 MBq ^44^Ti was used.

Unlike the Bio-Rad AG 1 × 8 anion exchange resin [20], in the case of TEVA resin, mixtures of 0.005 M H_2_C_2_O_4_/0.007 M HCl and 0.025 M H_2_C_2_O_4_/0.125 M HCl did not show satisfactory scandium-44 yield (despite the good Dg values obtained under static conditions). At the same time, eluents with compositions of 0.1 M H_2_C_2_O_4_/0.05–0.2 M HCl made it possible to achieve a sufficiently high yield of ^44^Sc. The breakthrough of ^44^Ti was about ≤5.5 × 10^−5^% when those compositions of eluent were used (except the very first elution directly after column loading). This corresponds to an exceptionally good separation factor of ≥1.8 × 10^6^.

Using pure 4.0 M HCl as eluent, almost quantitative (≥ 99 %) recovery of ^44^Ti from TEVA resin can be achieved (in 0.5 mL of eluate). No statistically significant difference in desorption rate of ^44^Ti from the columns that have been in use for 1 to 11 months was observed. This fact facilitating the regeneration and loading of new generator columns is also noteworthy.

**Figure 2:**
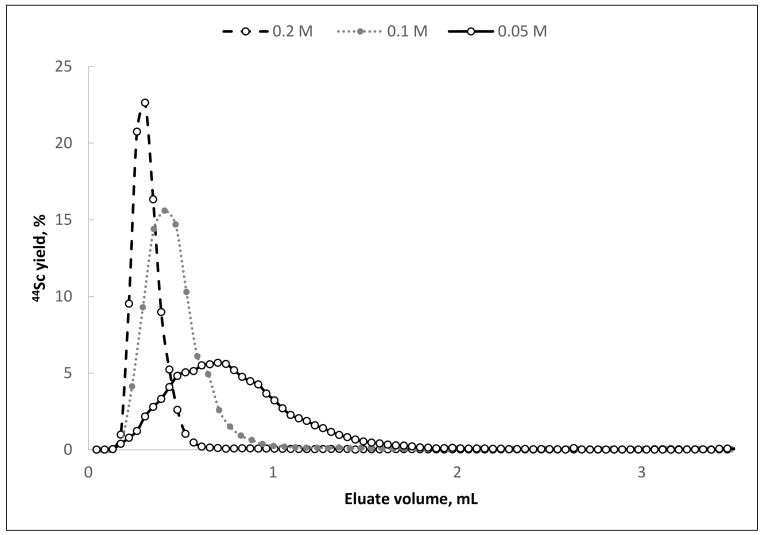
Elution curves of ^44^Sc from TEVA resin with eluents of various HCl concentrations (oxalic acid concentration—0.1 mol/L, flow rate—1 mL/min).

All three investigated HCl concentrations with 0.1 M oxalic acid content are attractive for further study. However, based on the elution profiles of the generator columns with different concentrations of hydrochloric acid (Figure 2), the mixture 0.1 M H_2_C_2_O_4_/0.2 M HCl (giving the highest average value of scandium-44 yield in the smallest eluate volume) was chosen to continue the study. This choice was made with the assumption that if, during long-term evaluation the behavior of titanium-44 will be satisfactory, then for eluents with a lower content of hydrochloric acid, the behavior of titanium-44 will be the same or better.

### 2.3. Elution Characteristics of TEVA-Based ^44^Ti/^44^Sc Generator Prototype (Long-Term Evaluation)

**Figure 3:**
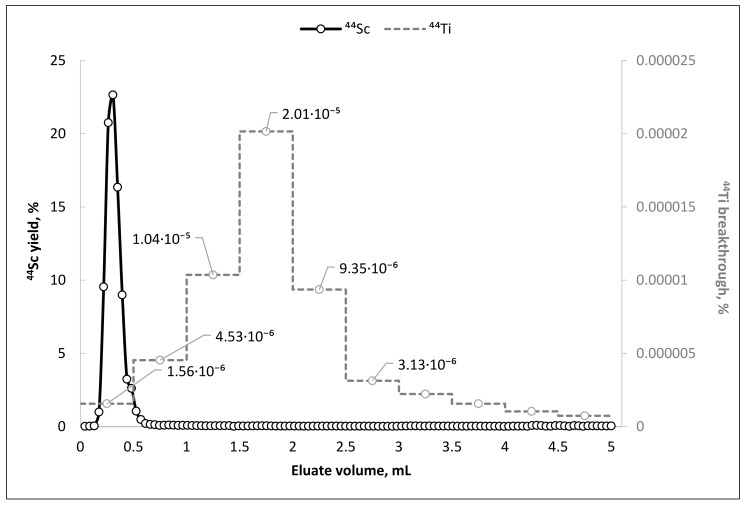
Typical elution curve of ^44^Sc from TEVA-based ^44^Ti/^44^Sc generator (primary axis) and values of ^44^Ti breakthrough in eluate fractions (0.5 mL each fraction; secondary axis), eluent—0.1 M H_2_C_2_O_4_/0.2 M HCl, flow rate—1 mL/min.

The 5.0 MBq TEVA-based ^44^Ti/^44^Sc generator prototype was used for long-term evaluation of its elution characteristics. The typical elution profile of ^44^Sc (as well as content of ^44^Ti in eluate fractions) are presented in Figure 3.

The experimental data show that, as in the case of pilot dynamic column separation experiments, elution of the main ^44^Sc activity (≥95% of all eluted activity) occurs in the first 0.5 mL. The ^44^Ti breakthrough strongly depends on the eluate volume and has a pronounced maximum (∼2 × 10^−5^%) at 1.5–2.0 mL fraction. This feature, in combination with the ability to elute scandium-44 in the first 1.0 mL of the eluate, makes it possible to achieve an extremely high separation factor of 1.6 × 10^7^.

**Figure 4:**
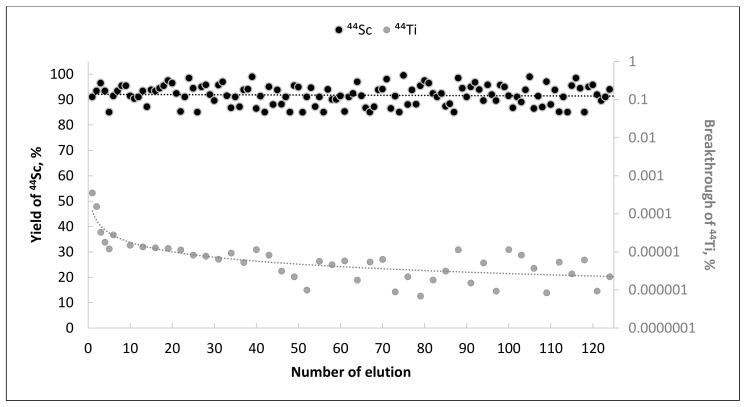
^44^Sc elution yield (primary axis) and ^44^Ti breakthrough (secondary axis) for TEVA resin generator prototype for over 100 elutions (eluate volume—1 mL, flow rate—1 mL/min, eluent—0.1 M H_2_C_2_O_4_/0.2 M HCl).

During 1 year of regular ^44^Sc elutions (at least 3 times a week, no more than 1 time a day), the ^44^Sc yield was 91 ± 6%, and the breakthrough of titanium-44 did not exceed 1.5 × 10^−5^% (Figure 4). The TEVA-based ^44^Ti/^44^Sc generator prototype had consistently high yields of ^44^Sc with no significant decreases with an increasing number of elutions. The ^44^Sc yield SD of 6% is due to the fact that elution was performed manually using a syringe and cannula, which, in turn, led to deviations in the eluent flow rate (decreases in the ^44^Sc yield are due to slightly faster elution rates; this aspect was subsequently fixed by the use of a syringe pump).

Only the very first eluate obtained directly after column loading contained relatively high amounts of ^44^Ti (3.5 × 10^−4^%). ^44^Ti breakthrough of 1.5 × 10^−4^% was obtained as soon as within the second elution. After 10 elutions, no more than 1.5 × 10^−5^% of ^44^Ti breakthrough was detected in the eluate. It is noteworthy that unlike other published ^44^Ti/^44^Sc generator prototypes (in the “direct” elution mode), there was a tendency towards decreasing the average value of ^44^Ti breakthrough for TEVA resin.

The autoradiography image of the ^44^Ti/^44^Sc generator column after 120 elutions showed main activity disposition in the top of the column (Figure S2), i.e., no dramatic spreading and blurring of ^44^Ti sorption zone in the column was observed for 1 year of testing.

**Figure 5:**
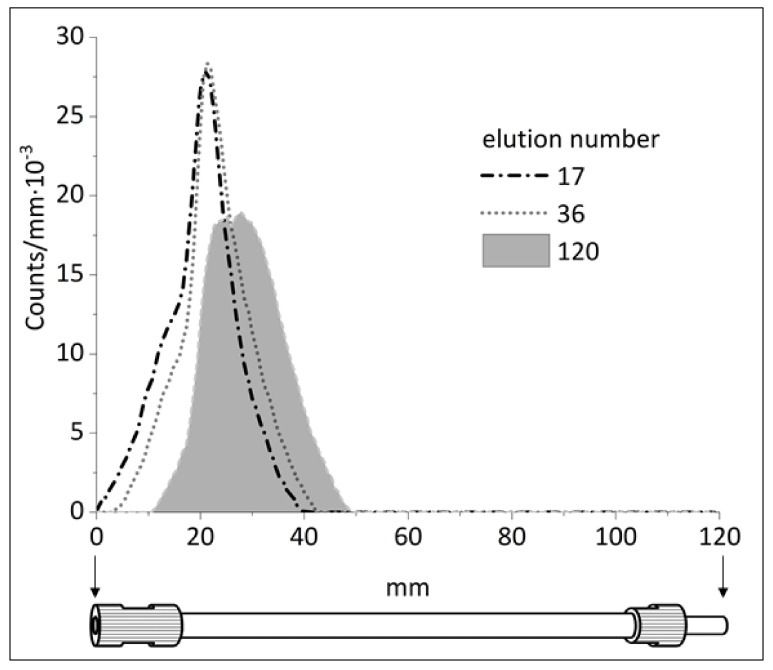
The activity distribution profiles of the generator column for increasing number of elutions.

The results of regular radiometry of the generator column demonstrates that some drifting of ^44^Ti sorption zone is nonetheless observed (Figure 5). Аfter 120 elutions, the maximum activity of the ^44^Ti zone shifted by almost 7 mm. Furthermore, after 120 elutions the full width at half maximum of the ^44^Ti zone increased from 13 to 19 mm.

Given that the column was eluted continuously only in direct mode, this shift of the ^44^Ti sorption zone still corresponds to good ^44^Ti retention on the resin. Based on the data on ^44^Sc elution yield and ^44^Ti breakthrough, as well as the absence of significant changes in the elution profile, the shift of ^44^Ti zone did not have any negative effect on the basic characteristics of the generator. The column is currently being monitored to establish the critical elution number after which the column will need to be regenerated.

### 2.4. Post-Elution Processing of Generator Eluate for Radiolabeling

For successful application in the field of radiopharmaceutical R&D, a generator should not only give satisfactory results in the separation of the parent and daughter radionuclides, but the resulting solution of the daughter radionuclide should also be suitable for the synthesis of RP with a high yield. The TEVA-based ^44^Ti/^44^Sc generator prototype has exceptionally good separation characteristics, but unfortunately its eluate is not suitable for direct use in radiopharmaceutical synthesis: after incubation of buffered eluate (pH 4.5, 1 mL) for 30 min at 95 °C with 50 nmol of PSMA-617 or PSMA-I&T precursors, no complex ([^44^Sc]Sc-PSMA-617 and [^44^Sc]Sc-PSMA-I&T) formation was observed. The high concentration of oxalic acid in the eluate interferes with the incorporation of scandium-44 into the structure of chelator-conjugated vector molecules. Thus, post-processing of the eluate is necessary for further use in radiopharmaceutical synthesis.

#### 2.4.1. Decomposition (Decarboxylation) of Oxalic Acid with H_2_O_2_ Followed by Evaporation

One of the classical approaches to remove oxalate from metal solutions, including solutions of n.c.a. metal radionuclides, is decarboxylation using hydrogen peroxide [25,26]. This method was successfully used, for example, in the case of ^89^Zr solutions [27], and was also proposed by Green in her work on the ^44^Sc generator [17].

After two evaporations of TEVA-based ^44^Ti/^44^Sc generator eluate (1 mL) with 500 μL of 30% H_2_O_2_, ^44^Sc was dissolved in 100 μL of 1.0 M HCl and then diluted to 1.0 mL with water (0.1 M HCl in final solution). As a result of this procedure, the concentration of residual oxalic acid in the final solution was 0.0001–0.0003 mol/L. The ^44^Sc recovery was about 87 ± 3%.

**Figure 6:**
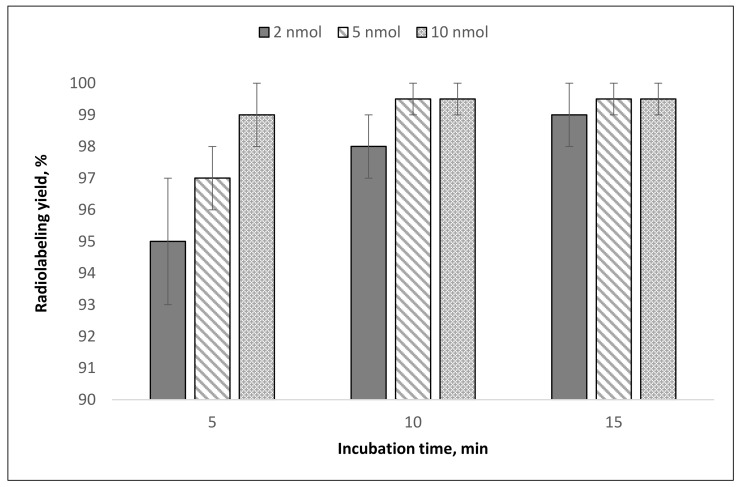
^44^Sc radiolabeling yields (mean ± SD, %; *n* = 7) for PSMA-617 at different precursor amounts and reaction times. Experimental conditions: convection heating 95 °C, V = 1.0 mL, pH = 4.5.

The results of [^44^Sc]Sc-PSMA-617 synthesis demonstrates that even with 2 nmol of precursor, a radiolabeling yield of >95% can be achieved within 15 min (Figure 6).

**Figure 7:**
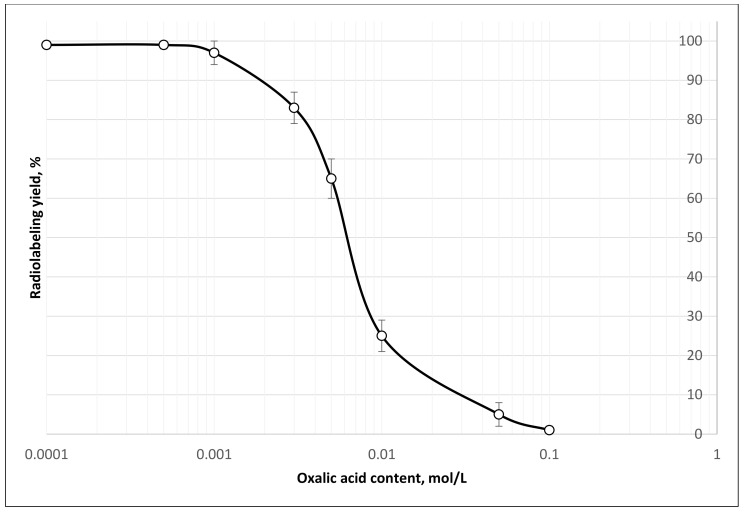
[^44^Sc]Sc-PSMA-617 radiolabeling yield depends on oxalate concentration in the reaction mixture (mean ± SD, %; *n* = 3). Experimental conditions: convection heating 95 °C, 30 min, V = 1.5 mL, pH = 4.5, 20 nmol PSMA-617.

Using these solutions of ^44^Sc spiked with oxalic acid, the maximum permissible concentration of oxalic acid for sufficient radiolabeling yield was determined (Figure 7). According to experimental data, for a radiolabeling yield of [^44^Sc]Sc-PSMA-617 higher than 95%, the content of oxalate anion in reaction mixture should be ≤0.001 mol/L. Even though the decarboxylation allows us to achieve good ^44^Sc-radiolabeling yields (it was also successfully used in a study on the complexation of ^44^Sc with oxabis(ethylenenitrilo)tetramethylenephosphonic acid [28]) this approach is not very technological and not very convenient for automatization and implementation in a synthesis module. It was decided to replace this approach with SPE chromatographic methods.

#### 2.4.2. Presep^®^ PolyChelate Resin

Various ion-exchange- and SPE-resins have been tested in search of one suitable for efficient trapping of ^44^Sc directly from the generator eluate (Table 3). Unfortunately, none of them showed satisfactory retention of scandium-44 in this case.

Considering the published results on the successful extraction of n.c.a. scandium radionuclides (^43,44,47^Sc) using chelating resins Chelex 100 [13,29,30,31] and NOBIAS Chelate-PA1 [32], as well as our results on the efficient isolation of medical radionuclides from oxalate solutions with Chelex 100 (e.g., ^89^Zr [33]), this type of resin has also been tested for the recovery of scandium-44 from the generator eluate.

Unfortunately, it turned out that Chelex 100 (iminodiacetic acid) is not capable of retaining scandium-44 from the generator eluate with sufficient efficiency (Table 4). NOBIAS Chelate-PA1 resin (iminodiacetic acid and ethylenediaminetriacetic acid) was not available for use. However, fortunately, we had a different resin at our disposal—Presep^®^ PolyChelate, а chelate resin immobilizing carboxymethylated pentaethylenehexamine [34,35]. Published data suggests that its performance is comparable to those of Nobias Chelate-PA1 and superior to those of some other commercially available aminocarboxilyc acid-type chelating resins [36,37].

Experiments under static conditions have shown that ^44^Sc distribution coefficient for this resin is: in 0.1 M HCl—3.8 ± 1.1 mL/g; in 0.2 M HCl—1.9 ± 0.7 mL/g; and for mixture of 0.1 M oxalic acid / 0.2 M HCl—>3 × 10^4^ mL/g.

Experiments under dynamic conditions (80 mg of the resin, column 50 mm × Ø2 mm, 1 mL/min) showed that ^44^Sc from the generator eluate is quantitatively trapped on this resin (sorption rate is ≥99%). To remove the residues of the sorption solution, the resin could be washed with both water and ethanol (1–5 mL). In both cases, the loss of activity during washing did not exceed 1%.

**Figure 8:**
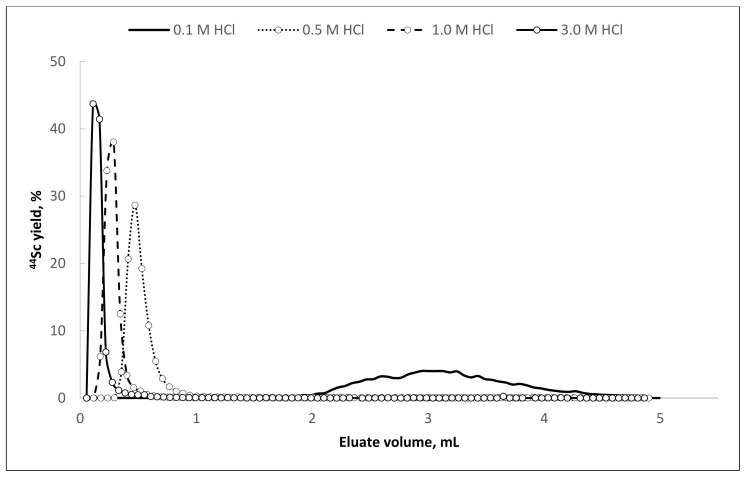
The elution curves of ^44^Sc from Presep^®^ PolyChelate resin with HCl of various concentrations as eluent (flow rate—1 mL/min).

It is noteworthy that ^44^Sc desorption can be performed using hydrochloric acid in the concentration range from 0.1 to 3.0 mol/L. With an increase of hydrochloric acid concentration in the eluent, ^44^Sc recovery rate increases, and the required volume of the eluate decreases (Table 5, Figure 8).

**Figure 9:**
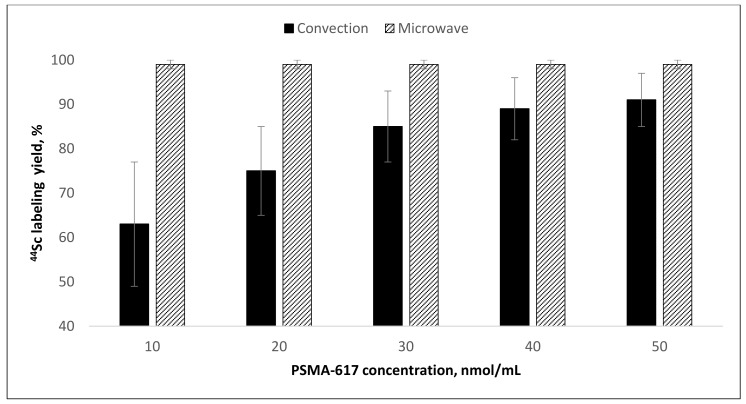
The dependence of ^44^Sc radiolabeling yields (mean ± SD, %; *n* = 5) for PSMA-617 on precursor amount and heating mode (after reformulation of ^44^Sc with Presep^®^ PolyChelate into 0.1 M HCl). Experimental conditions: 95 °C, 30 min, V = 1.0 mL, pH = 4.5.

Experimental data show that Presep^®^ PolyChelate resin is very promising for recovery of ^44^Sc from oxalate media, e.g., from TEVA-based ^44^Ti/^44^Sc generator eluate. At the same time, an unexpected fact was discovered: relatively high amounts of oxalic acid still remain present in the obtained ^44^Sc solutions. This amount is equal to the maximum permissible concentration of oxalic acid for sufficient radiolabeling and even exceeds it. Increased volumes of water and ethanol used for resin washing (till 10–15 mL) did not lead to significant improvements in residual oxalic acid content. The highest yield of [^44^Sc]Sc-PSMA-617 synthesis with ^44^Sc in 0.1 M HCl after Presep^®^ PolyChelate resin was about 92% (95 °C, 30 min, V = 1.0 mL, pH = 4.5, 20 nmol of PSMA-617). However, the average radiolabeling reaction yield at these conditions was only 75 ± 10%. It was found that microwave heating could significantly improve the radiolabeling reaction yield in this case (Figure 9).

**Figure 10:**
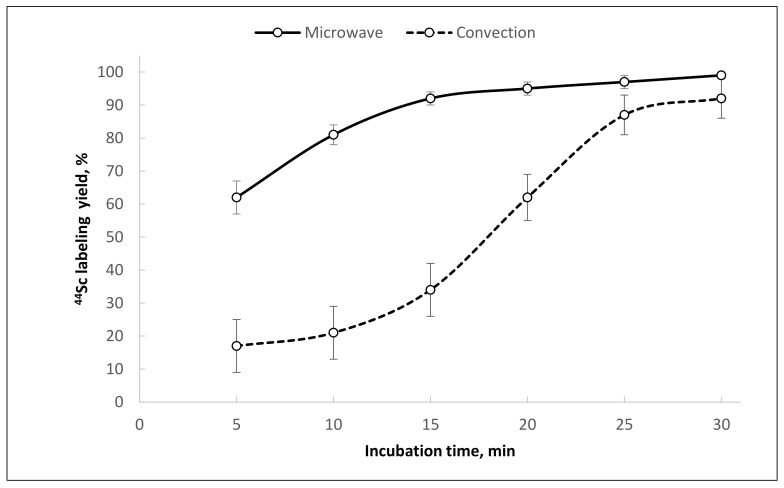
Kinetics of [^44^Sc]Sc-PSMA-617 formation using ^44^Sc after reformulation with Presep^®^ PolyChelate into 0.1 M HCl under different heating modes (95 °C, V = 1.0 mL, pH = 4.5, 50 nmol PSMA-617).

The use of microwave heating allowed a consistently high [^44^Sc]Sc-PSMA-617 synthesis yield (>95%) to be achieved in comparison to convective heating and stirring. This effect has already been demonstrated with ^68^Ga during synthesis of [^68^Ga]Ga-DOTATOC [38]. The kinetics of [^44^Sc]Sc-PSMA-617 formation indicate that even under microwave heating, the reaction is hampered and takes at least 25 min to reach sufficient yield (Figure 10).

Of particular interest is the fact that synthesis of [^44^Sc]Sc-PSMA-I&T under similar conditions (after reformulation of ^44^Sc with Presep^®^ PolyChelate into 0.1 M HCl, 95 °C, 30 min, V = 1.0 mL, pH = 4.5) gives consistently high yields (≥95%) with convective heating. The chelating agent DOTAGA (used in the structure of PSMA-I&T) appears to be less sensitive to the presence of chemical impurities compared to DOTA (used in the structure of PSMA-617) and much more readily forms complex with scandium-44 (Table 6).

**Figure 11:**
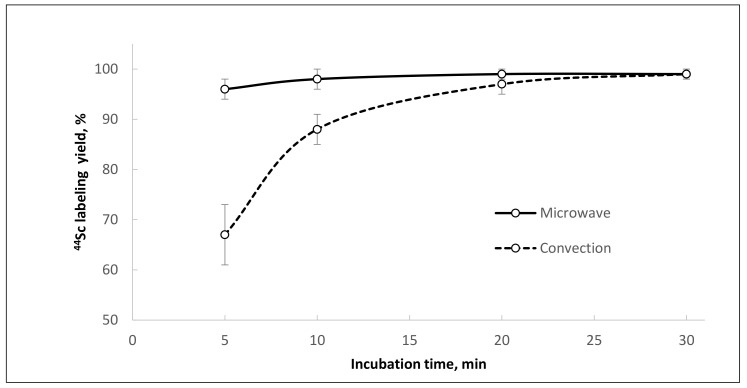
Kinetics of [^44^Sc]Sc-PSMA-I&T formation using ^44^Sc after reformulation with Presep^®^ PolyChelate into 0.1 M HCl under different heating modes (95 °C, V = 1.0 mL, pH = 4.5, 5 nmol PSMA-I&T).

Using ^44^Sc after reformulation with Presep^®^ PolyChelate into 0.1 M HCl and microwave heating for synthesis of [^44^Sc]Sc-PSMA-I&T achieved a labeling yield of >95% in only five minutes (with 5 nmol of precursor), and in 20 min with convective heating and stirring (Figure 11).

Since [^44^Sc]Sc-PSMA-I&T synthesis is less dependent on ^44^Sc solution quality, [^44^Sc]Sc-PSMA-617 labelling yield was further used to indicate its suitability. In this context, the solution obtained using Presep^®^ PolyChelate resin is much inferior to those obtained after decarboxylation. So, in this way an additional processing stage is required.

#### 2.4.3. Additional Reformulation Stage (Presep^®^ PolyChelate Resin + TK221 Resin)

The fact that ^44^Sc desorption from Presep^®^ PolyChelate resin can be carried out with hydrochloric acid of different concentrations (0.1–3.0 mol/L) opens a wide range of possible options for an additional purification stage, which may be either a cation exchange resin or a solid-phase extractant. In the course of this work, it was decided to test TK221 resin for this purpose [39,40].

Experiments under static conditions have shown that ^44^Sc distribution coefficients for TK221 resin in HCl media are as follows (mL/g): 0.01 M—24 ± 3, 0.1 M—33 ± 7, 0.5 M—41 ± 4, 1.0 M—61 ± 8, 2.0 M—1300 ± 200, 3.0 M—>1.5 × 10^4^. Experiments under dynamic conditions (80 mg of the resin, column 50 mm × Ø2 mm, 1 mL/min) showed that ^44^Sc in 3.0 M HCl after Presep^®^ PolyChelate resin is quantitatively trapped by TK221 resin (sorption rate is ≥99%).

As for desorption of scandium from TK221 resin it is necessary to use eluents with reduced acidity (mainly dilute acids according to the manufacturers data), several different eluents were investigated during this work (Table 7).

**Figure 12:**
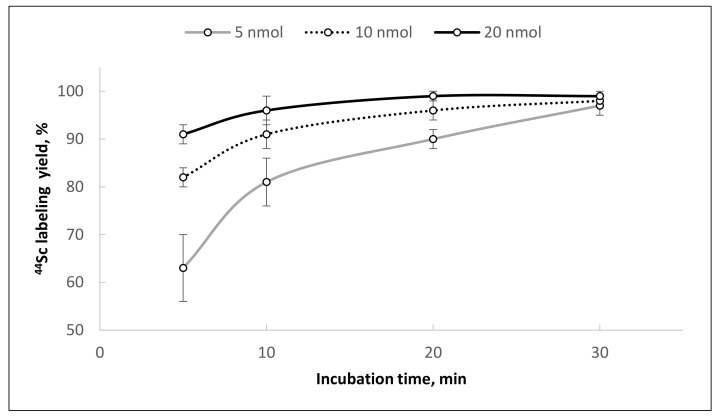
Kinetics of [^44^Sc]Sc-PSMA-617 formation using ^44^Sc after reformulation with Presep^®^ PolyChelate + TK221 reformulation and different precursor amounts (convective heating, 95 °C, V = 1.0 mL, pH = 4.5).

The highest ^44^Sc recovery was achieved with 1 M CH_3_COONH_4_ (0.5 mL, pH 4.5) after column flushing with a small (0.2 mL = 8 column bed volumes) volume of water. The characteristics of the final ^44^Sc solution are suitable for direct use in radioconjugate synthesis. The results of [^44^Sc]Sc-PSMA-617 synthesis demonstrate that a high radiolabeling yield (≥95%) can be achieved with 5 nmol of precursor after 30 min of incubation at 95 °C (V = 1.0 mL, pH = 4.5), and increasing the amount of PSMA-617 increased radiochemical yield (Figure 12).

**Figure 13:**
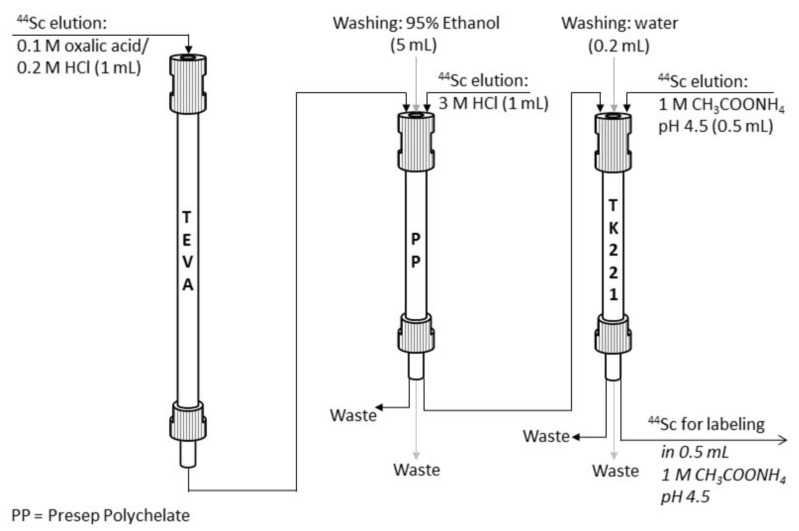
The scheme of proposed method for TEVA-based ^44^Ti/^44^Sc generator eluate post-processing.

The obtained data indicate that ^44^Sc solutions obtained by Presep^®^ PolyChelate + TK221 reformulation are suitable for synthesis of ^44^Sc-radioconjugates with sufficient yields. No ^44^Ti was detected in all samples of ^44^Sc solutions after combined post-processing. Thus, at this stage of our research, we can suggest that this method of TEVA-based ^44^Ti/^44^Sc generator eluate post-processing is preferable for ^44^Sc-radiopharmaceutical synthesis (Figure 13). The proposed method is simple to perform; it takes no more than 15 min (in manual mode), and the decay-corrected yield is ≥90% relative to the activity eluted from the generator.

## 3. Discussion

Today ^44^Sc seems to be an attractive and very promising radionuclide for small-molecule-based PET imaging. Growing interest in scandium-44 motivated research on different methods of its production, of course, mainly by using cyclotrons. However, development of the ^44^Ti/^44^Sc generator concept can be characterized by significant progress in recent years as well (Table 8).

After the first research by Greene [17], a 5 mCi ^44^Ti/^44^Sc radionuclide generator presented by Filosofov et al. [20], which was used in the first in-human use of [^44^Sc]Sc-PSMA-617 [10], became a real breakthrough in this area. An advanced generator concept involving alternating directions of elution flow, also proposed by Filosofov et al. [20], was an impressive solution. Only due to this, the characteristics of the generator reached the required values for further use. In the direct elution mode, the (0.1 M H_2_C_2_O_4_/0.2 M HCl) Bio-Rad AG 1 × 8 based generator results in increasing breakthrough of ^44^Ti with 50% desorption of ^44^Ti after 30 elutions, and an almost complete release of ^44^Ti after 50 elutions. In contrast to this, the TEVA-based ^44^Ti/^44^Sc generator, evaluated in this work, demonstrates high titanium-44 retention over 120 elutions with high ^44^Sc yield in small volumes and exceptionally low ^44^Ti breakthrough. Some drifting of ^44^Ti sorption zone is nonetheless observed. This ^44^Ti shift did not have any negative effect on the main characteristics of the generator so far. The generator column is currently being monitored.

Based on the experimental data under static and dynamic conditions obtained during this work, we believe that the use of increased column length, as well as an eluent with a lower hydrochloric acid concentration (0.1 M H_2_C_2_O_4_/0.1–0.05 M HCl instead of 0.1 M H_2_C_2_O_4_/0.2 M HCl) will significantly extend the generator’s shelf-life. These studies are being carried out by our team at the moment. Meanwhile, it is proposed that obtained separation characteristics for the TEVA-based ^44^Ti/^44^Sc generator demonstrate its ease of use and prospects for further work.

The investigation of described 5 MBq generator prototype may be considered as proof-of-concept for a TEVA-based ^44^Ti/^44^Sc generator. The radiolitic stability of TEVA and, therefore, the possibility to scale up ^44^Ti activity in the generator to at least 200 MBq remains to be addressed.

Considering all the difficulties associated with the presence of oxalic acid in the eluate and its negative effect on the radioconjugation efficiency (established in the course of this study), the ^44^Ti/^44^Sc generator proposed by Radchenko et al. [21] seems to be the most convenient for radiopharmaceutical purposes (since the eluent is 0.05 M HCl). At the same time, a ZR-based generator also requires alternating directions of elution flow, and ^44^Ti breakthrough in reverse mode exceeded that for Bio-Rad AG 1×8 and TEVA resins (although this was likely due to an off-center ^44^Ti loading activity placement). In only the direct elution mode did ^44^Ti breakthrough reach a value of 0.23% after ∼40 resin bed volumes. Thus, if we compare only the data for direct elution modes, TEVA-based generators provide the best retention of ^44^Ti. To our best knowledge, unfortunately, no additional studies have appeared with ZR-based ^44^Ti/^44^Sc generator so far. Additional comparison of ZR- and TEVA-based generators is also in our plans.

Radiolabeling experiments show that scandium-44 obtained from the generator is suitable for the synthesis of radiopharmaceuticals with high yield only after some post-processing because of the oxalic acid content. Apart from the problems with residual oxalate, a lucky find was that Presep^®^ PolyChelate resin captures scandium-44 quantitatively from the TEVA-based generator eluate. This resin looks very promising for recovery of ^44^Sc from oxalate solutions or similar media. The combination of Presep^®^ PolyChelate and TK221 resins made it possible to obtain ^44^Sc-radioconjugates with a high yield (≥95%), even with 5 nmol of precursor. Since ^44^Sc desorption from Presep^®^ PolyChelate resin can be carried out with hydrochloric acid of different concentrations, there is still a lot of room for improvement. For example, in addition to a variety of extraction resins, a cation exchange resin could be used if scandium-44 elution from Presep^®^ PolyChelate were performed with 0.1 M HCl. Or, finally, the eluate of the generator could be easily diluted to make scandium-44 trapping by cation exchange resin possible (by analogy with an already published method [41]).

Microwave heating of the reaction mixture considerably shortened the reaction time and improved radiolabeling yield and reproducibility. For [^44^Sc]Sc-PSMA-617, the radiochemical yield increased from 61 ± 14% to 98 ± 8% (10 nmol of precursor) with microwave heating, even at relatively high levels of oxalate anions. For [^44^Sc]Sc-PSMA-I&T, the labeling reaction was complete within 5 min (5 nmol of precursor), and the incorporation of ^44^Sc was almost quantitative (≥99%). The microwave heating method is still not convenient for radiopharmaceutical preparation, although its advantages in various aspects of radiochemical synthesis have already been shown [42,43,44]. Probably, in the future, with the expansion of production and organization of centralized supplies of radiopharmaceuticals, the use of microwave heating will take its rightful place.

The difference in the labeling efficiency of the DOTAGA-conjugated precursor (PSMA-I&T) over the DOTA-conjugated one (PSMA-617) is noteworthy. According to experimental data, the chelating agent DOTAGA appears to be less sensitive to the presence of chemical impurities compared to DOTA and much more readily forms a complex with scandium-44. Similar differences between DOTAGA- and DOTA-derivates of PSMA-ligands have also been demonstrated for ^177^Lu [45]. This fact should be considered in the development of new radiopharmaceuticals for diagnostics and therapy with radionuclides of Sc, Y, and the heaviest lanthanides.

## 4. Materials and Methods

### 4.1. Chemicals and Reagents

Only deionized water 18.2 MΩ·cm (Milli-Q Millipore, Merck, Darmstadt, Germany ) was used. All chemicals and solvents were of high purity or pharmaceutical grade. The chemicals were purchased from Sigma-Aldrich/Merck (St. Louis, MO, USA) or Panreac Quimica (Barcelona, Spain), unless otherwise indicated. The SPE resins TEVA (Aliquat^®^ 336) [46,47], TK221 (mixture of a diglocylamide and a phosphine oxide), UTEVA (diamyl, amylphosphonate, DAAP) [48], DGA (*N,N,N′,N′*-tetra-*n*-octyldiglycolamide) [49], and TRU (octylphenyl-*N,N*-di-isobutyl carbamoylphosphine oxide (CMPO) dissolved in tri-*n*-butyl phosphate (TBP)) [50] were kindly provided by TrisKem International (Bruz, France). The Presep PolyChelate resin was purchased from FUJIFILM Wako Pure Chemical Corporation (Osaka, Japan). The precursors PSMA-617 and PSMA-I&T were purchased from ABX advanced biochemical compounds GmbH (Radeberg, Germany) and were also kindly provided by Center of Molecular Research (Moscow, Russia).

### 4.2. Titanium-44

Titanium-44 as [^44^Ti]TiCl_4_ in 4.0 M HCl (36 MBq/mL, 30 MBq, specific activity >3.7×10^10^ Bq/g, metal impurities (μg/Bq): Sc < 1.05 × 10^−4^, Cu < 1.35 × 10^−5^, Fe < 2.07 × 10^−7^) was purchased from Cyclotron Ltd. (Obninsk, Russia).

### 4.3. Measurement of Radioactivity

Measurement of ^44^Sc radioactivity was accomplished using an Atomlab^TM^ 500 Dose Calibrator (Biodex, Shirley, NY, USA) and RIS-A1 (Amplituda Scientific and Technical Center, Moscow, Russia) dose calibrator. The relative activity (count rate) of the samples was measured with Wizard^2^ 2480 automatic γ-counter (PerkinElmer, Waltham, MA, USA). The absolute radioactivity of ^44^Sc and ^44^Ti was measured by γ-spectrometry using GR4020 spectrometer with a high-purity germanium detector (Mirion Technologies (Canberra) SAS, San Ramon, CA, USA) at γ-lines 511 and 1157.02 keV for both radionuclides being in secular equilibrium. Measurements of ^44^Ti breakthrough radioactivity as well as ^44^Ti distribution coefficients were accomplished using ultra-shielded γ-spectrometer with a highly-sensitive semiconductor detector GC10021 (Mirion Technologies (Canberra) SAS, (San Ramon, CA, USA) with a relative efficiency of 106.8% (^60^Co), FWHM 1.02 keV (^57^Co) and 2.03 keV (^60^Co)), with a DSA-1000 multichannel pulse amplitude analyzer, and Genie-2000 software (V3.2.1) at specific γ-lines 67.9 and 78.3 keV (with a control at 1157.02 keV) at the Laboratory of radiometric and spectroscopic research of human and environment (Burnasyan Federal Medical Biophysical Center). Measurements of ^44^Ti were performed at least after 300 h after samples preparation. The acquisition time for ^44^Ti breakthrough measurements was at least 24 h to achieve no less than 1×10^4^ full energy peak net counts in the regions of interest. A ^22^Na, ^44^Ti/^44^Sc, ^57^Co, ^60^Co calibration source (LLC “STC Amplituda”, Moscow, Russia) certified with k = 2.1 (Mendeleyev Institute for Metrology, St. Petersburg, Russia) was used. The breakthrough of ^44^Ti was calculated as the ratio of ^44^Ti activity in the generator eluate to its activity on the generator column.

Radiography of chromatographic columns (generator prototypes) and TLC-strips was carried out using PET-MiniGita radio-TLC scanner (Elysia-Raytest, Straubenhardt, Germany) as well as Cyclone^®^ Plus storage phosphor system (PerkinElmer, Waltham, MA, USA).

### 4.4. Distribution Coefficients

The mass distribution coefficient Dg was measured by the batch static method [51]. The sorption of ^44^Ti and ^44^Sc by resins from hydrochloric acid/oxalic acid solutions was measured by equilibration of a known volume (1 mL) of radionuclide spiked solution (80-500 kBq for ^44^Sc, and ∼12 kBq for ^44^Ti(^44^Sc)) of appropriate concentration with known weight of resin (typically 15 mg). Resins were weighed and used without preconditioning. The samples were mixed in the mechanical mixer with temperature control Bioer Mixing block MB-102 (Bioer, Hangzhou, China) for 24 h at 20 °C with stirring speed 1350 rpm. After that, the samples were centrifuged (Heidolph, Schwabach, Germany) for one minute at 15,000 rpm. The tubes were removed carefully (to avoid phase mixing). Then, the aliquots (100–500 μL) of every solution were taken and their relative activities were measured. The mass distribution coefficient (mL/g) was defined according to the following Equation (1):

(1) Dg=A0−AA×VmLmg

where *A*_0_—count rate of the solution aliquot before the contact with the resin (decay corrected); *A*—count rate of the solution aliquot after the contact with the resin (decay corrected); *V*—solution volume, mL; *m*—weight of dry resin, g. Every Dg coefficient was determined at least in 5 parallel experiments.

### 4.5. Dynamic Column Separation Experiments and ^44^Ti/^44^Sc Generators Preparation

Evaluation of the ion exchange behavior of ^44^Sc and ^44^Ti in dynamic conditions was performed using PEEK chromatographic columns 100 mm × Ø2.1 mm and 50 mm × Ø2.1 mm (VICI Jour, Schenkon, Switzerland). Transfer of solutions was performed using syringes (BBraun Injekt) manually or with a syringe pump.

For ^44^Ti/^44^Sc generators, only 100 mm × Ø2.1 mm columns were used. The columns were filled with TEVA resin (148–150 mg per column) and stuffed with PEEK-encased polyethylene frits and 1/4–28 female to Luer PEEK adapters. A sample of ^44^Ti solution in 4.0 M HCl with desired activity was evaporated to dryness, and ^44^Ti was taken up with 150 μL of 0.1 M oxalic acid. The resulting solutions were slightly opalescent, so 5 μL of 1.0 M HCl was added to make them transparent. The obtained solutions were transferred quantitatively to generator columns (using 1–2 additional portions (150 μL) of 0.1 M oxalic acid). The elution of the generators was carried out with the selected solutions of hydrochloric and oxalic acids at least 3 times a week, no more than 1 time a day. Elution was carried out fractionally (each fraction—1 mL, total elution volume—1–5 mL) except the experiments when elution curves were determined (each fraction—45–50 μL).

### 4.6. Radiolabeling of PSMA-617 and PSMA-I&T

Radiolabeling of PSMA-617 and PSMA-I&T with ^44^Sc was carried out at pH 4.5 using 1.0–2.0 M CH_3_COONH_4_ as buffer. In the case of convective heating, the reaction was performed using a 2.0 mL Eppendorf test tube and Bioer Mixing block MB-102 (Hangzhou, China) at 95 °C (5–30 min). In the case of microwave heating, the reaction was performed with CEM Discover 908005 Microwave Synthesis System in Biotage^®^ Microwave Reaction Vials (Uppsala, Sweden) of 0.5–2 mL (95 °C, 5–30 min). The necessary amount of precursor (2-50 nmol) was mixed in the tube/vial with the ^44^Sc solution (∼1.0–4.0 MBq of ^44^Sc in 0.1 M HCl or in 1.0 M CH_3_COONH_4_, рН 4.0–4.5). If needed, the required pH was achieved by adjustment with 3.0 M HCl. The CH_3_COONH_4_ concentration in the reaction mixture was kept at a level of 1.0 mol/L, and the final volume of the reaction mixture was 1 mL.

A number of radio TLC methods were used to analyze the radiochemical purity (radiochemical yield) of the preparations obtained. The main TLC methods were: (a) ITLC-SG paper (Agilent, Santa Clara, CA, USA) with 0.05 M citric acid water solution as solvent; (b) ITLC-SG paper with 1 M CH_3_COONH_4_ in methanol–water mixture (1:1) as solvent; and (c) silica gel coated aluminum TLC plates (5553, Merck, Darmstadt, Germany) with acetonitrile-water mixture (1:1) as solvent. In method (a) the *Rf* of unbounded ^44^Sc is 0.9–1.0, and the *Rf* of [^44^Sc]Sc-PSMA-617 and [^44^Sc]Sc-PSMA-I&T is 0.0-0.1. In method (b), the *Rf* of unbounded ^44^Sc is 0.0–0.1, and the *Rf* of [^44^Sc]Sc-PSMA-617 and [^44^Sc]Sc-PSMA-I&T is 0.8–1.0. In method (c), the *Rf* of unbound ^44^Sc is 0.0–0.1 and the *Rf* of [^44^Sc]Sc-PSMA-617 and [^44^Sc]Sc-PSMA-I&T is 0.9–1.0. For HPLC analysis, one method was used. In short: a Knauer Smartline HPLC system (Berlin, Germany) equipped with an fLumo radiometric detector (Berthold, Bad Wildbad, Germany) and reversed phase C_18_ column (250 × 4.6 mm column; Jupiter, Phenomenex Inc.,) was used. The column thermostat temperature was 55 °C, gradient flow (0.85 mL·min^−1^): 0–13–13.5–21 min = 85–28–85–85 % A (A—0.1% TFA_aq._, B—methanol). Retention time using this method for [^44^Sc]Sc-PSMA-617 and [^44^Sc]Sc-PSMA-I&T was 13.2 ± 0.3 min, and for [^44^Sc]Sc^3+^ was 4.1 ± 0.2 min.

### 4.7. Determination of Oxalic Acid

Determination of oxalic acid content in hydrochloric acid samples was accomplished using HPLC. LC-20AD Prominence chromatograph (Shimadzu Co. Ltd., Kyoto, Japan) was used. The analysis was conducted on a Shodex 1C SI-90 4E column (4.0 mm Ø × 250 mm, Shodex Group, Tokyo, Japan). Detection was performed using diode array detector SPD-M20A at 210 nm. The column thermostat temperature was 33 °C, isocratic flow (1.5 mL·min^−1^): 1.7 mM NaHCO_3_ + 1.8 mM Na_2_CO_3_. Retention time for oxalate anion using this method is 28.2 ± 0.2 min.

## 5. Conclusions

Based on the results obtained in different studies, to date it is believed that ^44^Sc has significant clinical potential for PET imaging as a diagnostic match to therapeutical radionuclides of rare-earth elements (e.g., ^47^Sc, ^90^Y, ^153^Sm, ^161^Tb, ^177^Lu) in the concept of theragnostic approaches. The more scientific groups involved in the development of the radiopharmaceutical chemistry of scandium, the easier and faster it will be to introduce it into routine clinical practice. From this point of view, evaluation of the ^44^Ti/^44^Sc generator (and in particular the TEVA-based ^44^Ti/^44^Sc generator) will allow scientific groups, deprived of a cyclotron, to begin their work with ^44^Sc-radioconjugates. We also hope that our data on the efficacy of TEVA resin will stimulate the development of new extractants suitable for titanium-44 retention with improved efficiency.

## Figures and Tables

**Table 1:** Distribution coefficients (Dg) of ^44^Ti and ^44^Sc in various H_2_C_2_O_4_/HCl mixtures for TEVA resin.

Liquid Phase Composition		Dg, mL/g	
Oxalic Acid, mol/L	HCl, mol/L	^44^Ti	^44^Sc
0	4	0.31	1.27
0.1	0	7.2 × 10^4^	2800
	0.01	6.1 × 10^4^	25.1
	0.05	4.7 × 10^4^	9.3
	0.1	3.6 × 10^4^	4.9
	0.2	2.2 × 10^4^	2.7
	0.5	6800	2.1
	1.0	2200	1.3
0.025	0.125	1.8 × 10^4^	6.8
0.005	0.007	1.1 × 10^4^	24.5

**Table 2:** Dependence of ^44^Sc yield on eluent composition (mean ± SD, n = 10).

Eluent Composition		Elution Volume, mL	^44^Sc Yield, %
Oxalic Acid, mol/L	HCl, mol/L		
0.1	0.05	5	90 ± 3
	0.1		91 ± 3
	0.2		93 ± 4
0.025	0.125		21 ± 4
0.005	0.007		13 ± 3

**Table 3:** The results of ^44^Sc sorption on different resins from TEVA-based ^44^Ti/^44^Sc generator eluate.

Resin Type	Amount of Resin in Column, mg	Sorption of ^44^Sc, %
Сation Exchange Resins		
Chromafix HR-XC	80 ± 5	35–55
Sep-Pak Plus light CM		32–40
Dowex 50 W × 4		37–45
OASIS MCX		49–53
Purelite Сhromalite 50 × 2		37–45
Strata XC		48–57
SPE resins		
TRU	70 ± 5	12–15
TK200		8–10
DGA		13–20
TBP		11–15
UTEVA		9–12

**Table 4:** Sorption of ^44^Sc from TEVA-based ^44^Ti/^44^Sc generator eluate on different chelating resins.

Resin Type	Amount of Resin in Column, mg	Sorption of ^44^Sc, %
Chelex 100	80 ± 5	13 ± 1
Lewatit MonoPlus TP 207		26 ± 2
Presep PolyChelate		≥99

**Table 5:** Desorption of ^44^Sc from Presep^®^ PolyChelate resin with HCl of different concentration (mean ± SD, %; *n* = 5).

HCl Concentration in Eluent, mol/L	Desorption of ^44^Sc, %	Eluate Volume, mL	Concentration of Residual Oxalic Acid in Eluate, mol/L
0.1	87 ± 3	5.0	0.001–0.005
0.5	89 ± 2	1.5	0.005–0.008
1.0	93 ± 3	1.0	0.008–0.010
3.0	95 ± 4	0.5–1.0	0.008–0.010

**Table 6:** The yield of ^44^Sc radioconjugate formation using different amounts of precursors (mean ± SD, %; *n* = 5; 95 °C, 30 min, V = 1.0 mL, pH = 4.5).

Radioconjugate	Heating Mode	Precursor Amount, nmol				
		10	20	30	40	50
**[^44^Sc]Sc-PSMA-617**	microwave	98 ± 2	98 ± 1	≥99	≥99	≥99
	convection	61 ± 14	75 ± 10	85 ± 8	89 ± 7	91 ± 6
**[^44^Sc]Sc-PSMA-I&T**	microwave	≥99	≥99	≥99	≥99	≥99
	convection	98 ± 2	98 ± 2	98 ± 2	99 ± 1	99 ± 1

**Table 7:** The rate of ^44^Sc desorption from TK221 resin with different eluents and resin washing conditions.

Eluent	Column Wash before Elution	Desorption of ^44^Sc, %	Eluate Volume, mL
H_2_O	-	7 ± 4	1
0.05 M HCl	-	34 ± 2	1
0.1 M HCl	-	31 ± 3	1
0.1 M HCl	5 M NaCl in 0.1 M HCl (1 mL)	90 ± 4	3
1 M CH_3_COONH_4_, рН = 7.0	-	91 ± 2	1
1 M CH_3_COONH_4_, рН = 7.0	3 M HCl (1 mL)	35 ± 3	1
1 M CH_3_COONH_4_, рН = 4.5	H_2_O (0.2 mL)	97 ± 2	0.5

**Table 8:** Characteristics of various chromatographic ^44^Ti/^44^Sc generators reported so far.

Sorbent	Eluent	Loaded ^44^Ti Activity, MBq	Eluate Volume, mL	*Elution Mode*		Ref.
				^44^Sc Yield, %	^44^Ti Breakthrough, %	
Dowex 1 × 8	0.1 M oxalic acid /0.2 M HCl	Not specified	30–50	*Direct*		[17]
				60–70	0.02 → 0.1 (*after 40 elutions*)	
ZrO_2_·nH_2_O	0.01 M HCl	0.037	30	*Direct*		[19]
				42–46	0.02	
Bio-Rad AG 1 × 8	0.005 M oxalic acid /0.07 M HCl	185	20	*Reversed*		[20]
				97	5 × 10^−5^	
ZR resin	0.05 M HCl	3.7	5	*Reversed*		[21]
				Not clearly specified	Forward direction—no detectable, Reverse direction—4.1 × 10^−4^	
TEVA resin	0.1 M oxalic acid /0.2 M HCl	5.0	1	*Direct*		This work
				91 ± 6	≤1.5 × 10^−5^ (Normally 6.6 × 10^−6^)	

## Data Availability

The data presented in this study are available on request from the corresponding author. The data are not publicly available in accordance with current rules of Federal Medical Biological Agency of Russia for research carried out under state assignment.

## Supplementary Materials

The following are available online, Figure S1: Chemical structures of PSMA-617 and PSMA-I&T, Figure S2: The generator column radiography. From left to right: the column photo; the column autoradiography image; the column combined image.

## Sample Availability

Samples of the compounds are not available from the authors.
