# Supplementary material for: Separation of 44Sc from 44Ti in the Context of A Generator System for Radiopharmaceutical Purposes with the Example of [44Sc]Sc-PSMA-617 and [44Sc]Sc-PSMA-I&T Synthesis
Source: Molecules. 2021 Oct 21;26(21):6371. doi: 10.3390/molecules26216371 (PMC8587778; doi:10.3390/molecules26216371)
Supplement: Supplementary file 1 [file molecules-26-06371-s001.zip › molecules-1413560-supplementary.pdf]

Supplementary materials

Separation of ^{44}Sc from ^{44}Ti in the context of generator system for radiopharmaceutical purposes with example of [^{44}Sc]Sc-PSMA-617 and [^{44}Sc]Sc-PSMA-I&T synthesis

Anton A. Larenkov *, Artur G. Makichyan and Vladimir N. Iatsenko

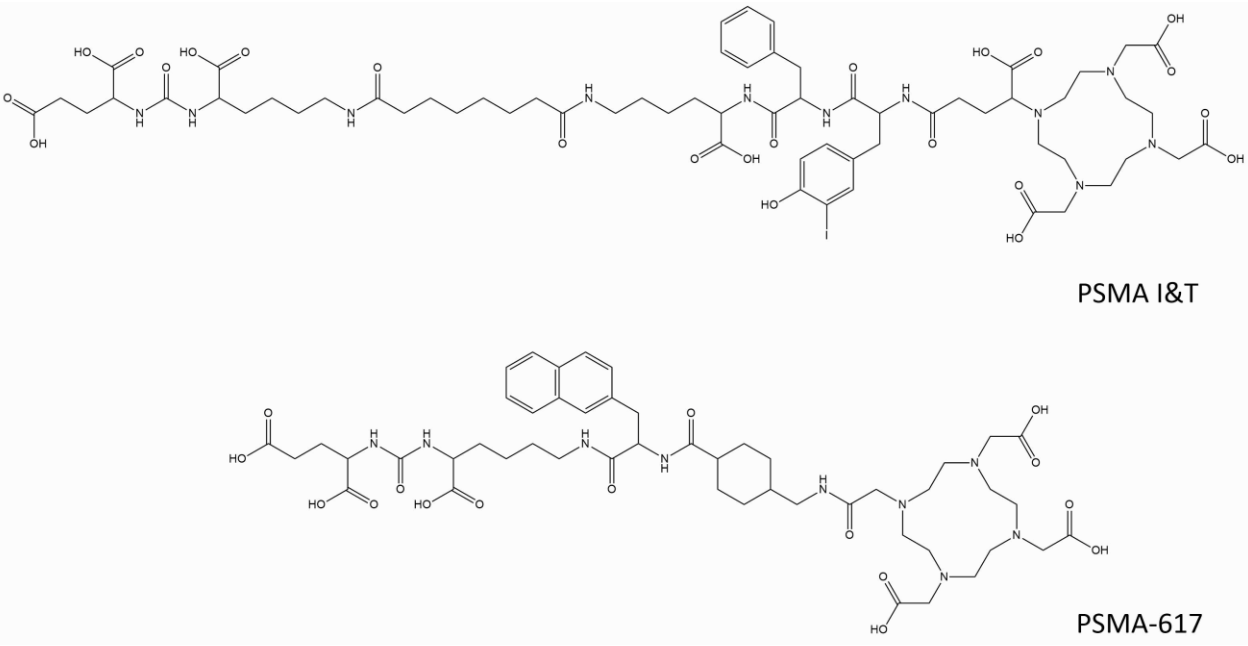

Figure S1. Chemical structures of PSMA-617 and PSMA-I&T.

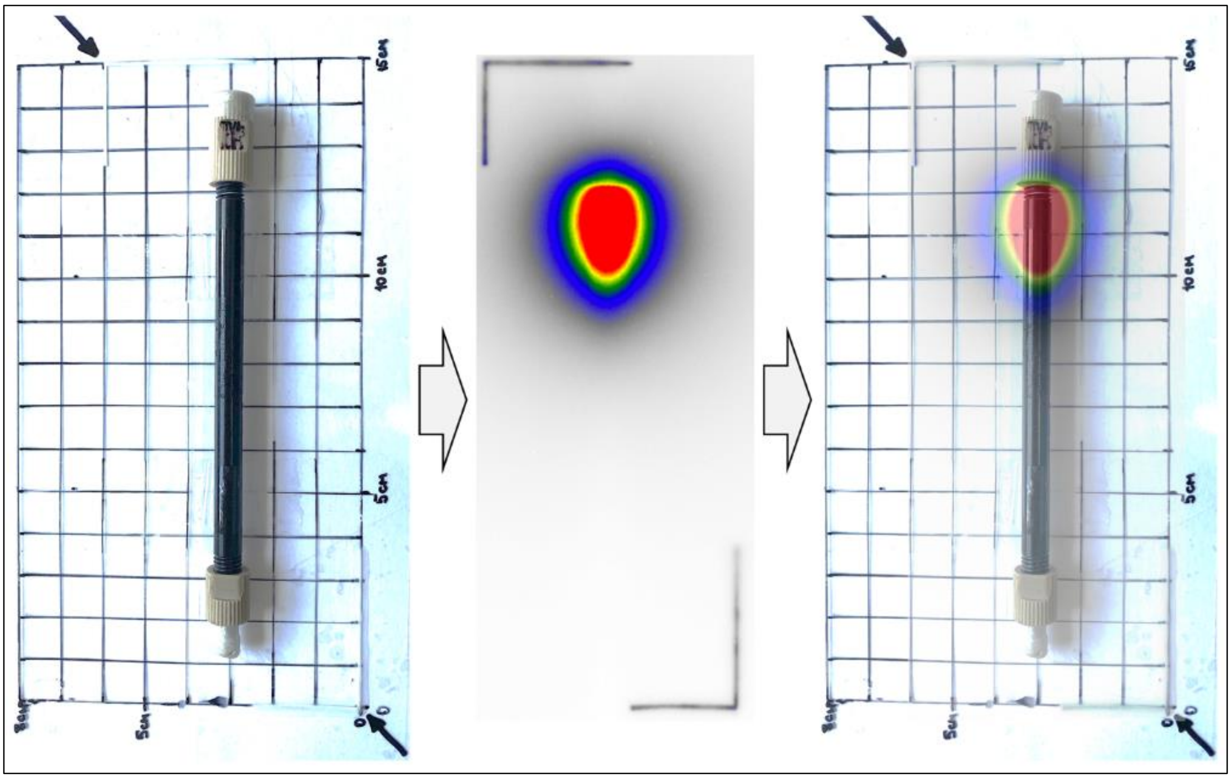

Figure S2. The generator column radiography. From left to right: the column photo; the column autoradiography image; the column combined image.
